# Cognitive Dysfunction in Asian Patients with Depression (CogDAD): A Cross-Sectional Study

**DOI:** 10.2174/1745017901713010255

**Published:** 2017-12-05

**Authors:** Srisurapanont Manit, Mok Yee Ming, Yang Yen Kuang, Chan Herng-Nieng, Della Constantine D, Zainal, Nor Zuraida, Jambunathan Stephen, Amir Nurmiati, Kalita Pranab

**Affiliations:** 1 Department of Psychiatry, Faculty of Medicine, Chiang Mai University, Chiang Mai, Thailand; 2 Institute of Mental Health, View, Buangkok Green Medical Park, Buangkok, Singapore; 3 Department of Psychiatry, National Cheng Kung University, Cheng Kung National University Hospital, Tainan, Taiwan,; 4 Department of Psychiatry, Singapore General Hospital, Academia, Singapore; 5 College of Medicine, University of the Philippines, Manila, Philippines; 6 University Malaya, Jalan Universiti, Kuala Lumpur, Wilayah Persekutuan, Malaysia; 7 Department of Psychiatry, Cipto Mangunkusumo Hospital, University of Indonesia, Jakarta Pusat, Indonesia; 8 Lundbeck Singapore Pte. Ltd., 101 Thomson Road, Singapore

The correct spelling of last author's name is provided and replaced online which is mentioned as under:

Srisurapanont Manit, Mok Yee Ming, Yang Yen Kuang, Chan Herng-Nieng, Della Constantine D, Zainal, Nor Zuraida, Jambunathan Stephen, Amir Nurmiati, Kalita Pranab

The wrong spelling was: 

Srisurapanont Manit, Mok Yee Ming, Yang Yen Kuang, Chan Herng-Nieng, Della Constantine D, Zainal, Nor Zuraida, Jambunathan Stephen, Amir Nurmiati, Kalita Pranabi

